# Glucosamine supplementation during late gestation alters placental development and increases litter size

**DOI:** 10.1186/s40104-017-0198-9

**Published:** 2017-09-01

**Authors:** Jeffrey L. Vallet, Jeremy R. Miles, Bradley A. Freking, Shane Meyer

**Affiliations:** 1 USDA, ARS, U.S. Meat Animal Research Center (USMARC), P.O. Box 166, Clay Center NE, Nebraska, 68933 USA; 2 Plymouth Ag Group, Diller NE, Nebraska, 68342 USA

**Keywords:** Fructose, Glucosamine, Hyaluronan, Swine, Uterine capacity

## Abstract

**Background:**

During late gestation the placental epithelial interface becomes highly folded, which involves changes in stromal hyaluronan. Hyaluronan is composed of glucoronate and N-acetyl-glucosamine. We hypothesized that supplementing gestating dams with glucosamine during this time would support placental folded-epithelial-bilayer development and increase litter size. In Exp. 1, gilts were unilaterally hysterectomized-ovariectomized (UHO). UHO gilts were mated and then supplemented daily with 10 g glucosamine (*n* = 16) or glucose (control, *n* = 17) from d 85 of gestation until slaughter (d 105). At slaughter, the number of live fetuses was recorded and each live fetus and its placenta was weighed. Uterine wall samples adjacent to the largest and smallest fetuses within each litter were processed for histology. In Exp. 2, pregnant sows in a commercial sow farm were supplemented with either 10 g glucosamine or glucose daily from d 85 of gestation to farrowing. Total piglets born and born alive were recorded for each litter. In Exp. 3, the same commercial farm and same protocol were used except that the dose of glucosamine and glucose was doubled to 20 g/d.

**Results:**

In Exp. 1, the number of live fetuses tended to be greater in glucosamine-treated UHO gilts (*P* = 0.098). Placental morphometry indicated that the width of the folded bilayer was greater (*P* = 0.05) in glucosamine-treated gilts. In Exp. 2, litter size did not differ between glucosamine- and glucose-treated sows. However in Exp. 3, the increased dose of glucosamine resulted in a significant treatment by parity interaction (*P* ≤ 0.01), in which total piglets born and born alive were greater in glucosamine treated sows of later parity (5 and 6).

**Conclusions:**

These results indicated that glucosamine supplementation increased the width of the folds of the placental bilayer and increased litter size in later parity, intact pregnant commercial sows.

## Background

Litter size contributes to the profitability of swine production, and is influenced by ovulation rate, fertilization rate, embryonic mortality and uterine capacity [1]. Fertilization rate and embryonic mortality are typically fixed rates that are independent of oocyte or embryo number [1, 2]. Thus, a greater ovulation rate results in increased embryos at d 30 of gestation [3]. However, greater ovulation rate does not result in greater litter size at farrowing [4], but does reduce birth weight [3]. In addition, conceptus losses under crowded uterine conditions occur throughout gestation [3, 5]. Both phenomena are the result of reduced placental size caused by intrauterine crowding.

Recent studies suggest that the pig placenta compensates for reduced intrauterine space [6]. A component of this adaptation is likely to be increased depth of the microscopic folds of the placental epithelial bilayer [7]. However, Vallet and Freking [7] also reported that placenta of small fetuses lacked stromal tissue above the folded bilayer, especially during late gestation, potentially limiting the compensatory ability of the placenta. Hyaluronan is a major component of placental stroma [8, 9] and is composed of repeating units of N-acetyl glucosamine and glucuronate, which are both derivatives of glucose [10]. Interestingly, Glucose transporter (GLUT) 2, which is present at the fetal maternal interface [11], has greater transport capacity for glucosamine compared to glucose [12]. We hypothesized that supplementation of glucosamine in sow diets might preferentially promote placental stromal development, allowing increased placental epithelial bilayer fold development. This would increase uterine capacity of glucosamine-treated sows. The objective of these experiments was to test whether glucosamine supplementation alters placental fold development, uterine capacity and litter size.

## Methods

### Experiment 1

Gilts were unilaterally hysterectomized-ovariectomized (UHO) at 160 d of age. Gilts were anesthetized with sodium pentothal and anesthesia was maintained using fluothane. The UHO surgery involves removing one ovary and one uterine horn, and reduces the total intrauterine space by one-half, while the ovulation rate remains unaffected. The litter size of pigs after UHO is no longer affected by ovulation rate, and is considered to be a measure of one-half their uterine capacity [5]. Gilts were fed 2 kg/d of a diet that met NRC requirements for pigs, consisting of 70% ground corn, 25% soybean meal with the remainder made up of vitamin and mineral supplements, free lysine and soybean oil. Gilts were allowed to recover and were subsequently naturally mated to mature boars after at least one estrous cycle of normal length (17 to 23 d). Thirty-three pregnant UHO gilts were used in this experiment. Beginning at d 85 of gestation, gilts were fed in individual pens and received either 10 g glucosamine (Hard Eight Nutrition LLC, Henderson NV) or 10 g glucose (Pastry Chef Central Inc., Boca Raton FL) as a top dress on their daily feed. The dose of glucosamine was chosen to be similar to that routinely used for supplementation in humans, after accounting for differences in weight (1.5 to 3 g per day are recommended in adult humans). Supplementation on d 85 of gestation was chosen because secondary fold development begins at this time [13]. Secondary fold development requires significant stromal remodeling and hyaluronan turnover. Because hyaluronan is 50% glucosamine, we hypothesized that glucosamine supplementation would facilitate fold development by supporting hyaluronan turnover. At 105 d of gestation, gilts were humanely slaughtered and the remaining uterine horn and ovary were collected. Corpora lutea were counted, and the umbilical cord of each live fetus was exteriorized through a small antimesometrial hole in the uterine wall to minimize disruption of the placental vasculature. Then, blood samples were taken from each fetus. A fetus was considered alive if it had a visible pulse in the exposed umbilical cord. Blood samples were taken from the umbilical artery of each live fetus. The remaining uterine horn was then opened completely and each live fetus was counted and weighed. The largest and smallest fetuses by weight were identified, their corresponding placentas were identified, and a uterine wall sample was collected that included these placentas. The smallest live fetus in each litter was chosen as this would be the most compromised fetus within the UHO litter. It was compared to the largest fetus in the litter as this would be the most uncompromised fetus in the litter. Thus, sampling the largest and smallest fetuses represented the full range of weight variation within each litter. Uterine samples were taken immediately adjacent but external to the amnion. Tissues were placed into cassettes and immersed in buffered formalin. Finally, a further sample of fetal placental tissue was collected and frozen in liquid nitrogen.

After formalin fixation, uterine wall samples were transferred to 70% ethanol in water, dehydrated through a series of increasing alcohol concentrations followed by xylene, and then embedded in paraffin. Tissues were then sectioned (10 μm), placed on slides, rehydrated and stained with hematoxylin and eosin, dehydrated and coverslipped. At least two sections were evaluated for each placental sample. Width of the folded bilayer, width of the stroma above the folds, and the interface length adjusted to constant placental length were measured using Bioquant (Bioquant image analysis corporation, Nashville, TN) as previously described [7], includes figure describing individual measures. Briefly, to obtain these measures, the area of a folded region within the placental interface was obtained by creating a closed polygon that extended from the base of the first fold to its top, across the top of several (3 to 5) adjacent folds, from the top to the bottom of the last fold, and across the bottom of the adjacent folds. Then, the length of the polygon along the long axis of the folded bilayer was measured by drawing a line through the center of the folded region within the polygon. The average width of the polygon (width of the folded bilayer) was calculated as the area of the polygon divided by its length. The width of the stroma was measured from the tip of each fold within the polygon to the adjacent edge of the stroma (border of the allantois). These measures were averaged for each slide to provide a single stromal width measure for each slide. Finally, the adjusted placental interface length was obtained by measuring (i.e., tracing) the length of the folded bilayer within the polygon, and dividing that length by the length of the polygon through the center. Each of the three measures was then averaged for the two slides for each placenta to provide a single measure of the width of folds, width of stroma and adjusted placental interface length for each placenta.

Fetal blood samples were allowed to clot, centrifuged, and serum was collected. Serum samples from each fetus were measured for glucose and fructose. Glucose was measured using the YSI 2700 Biochemistry Analyzer (YSI Life Sciences, Yellow Springs, OH) using instructions included in the manual. Fructose was measured using the procedure described by Zavy et al. [14].

Placental tissue samples were homogenized as described by Vallet et al. [9] and homogenates were measured for hyaluronan using a kit (Corgenix Inc., Broomfield CO). Homogenized placental samples were diluted 1:500 in PBS before assay.

### Experiment 2

This trial took place at a commercial sow farm in Nebraska (Plymouth Ag Group, Diller, NE) in May–July, 2015. The farm farrowed approximately 255 sows over a two-week period in weekly batches. Sow parity in both weeks ranged from 2 to 8. Sows were managed (including diet) and bred by artificial insemination according to the normal protocols existing at the farm, and were then supplemented with either glucosamine (*n* = 128; 10 g/d) or glucose (*n* = 127; control, 10 g/d) as a top dress on their daily feed (~2 kg corn-soybean diet depending on body condition) beginning on d 85 of gestation. Care was taken to evenly distribute glucosamine or glucose treatment among parities. Top dress was delivered using plastic scoops previously calibrated to deliver the appropriate amounts of glucosamine or glucose. During gestation, sows were housed in individual gestation stalls according to the standard procedure for the farm, allowing for them to be dosed independently on a daily basis. At d 115 of gestation, sows received an injection of estrumate (cloprostenol; Merck Animal Health, Madison, NJ) to induce farrowing, which is also standard procedure for the management of farrowing on the farm. Number born, number born alive, number stillborn, number of mummified fetuses, and the number of piglets weaned were recorded for each litter. All live piglets were ear tagged at birth, and birth and weaning weights for each live piglet were also recorded for each litter. Piglets were crossfostered according to procedures used on the commercial farm, but it was not possible to record piglet movement except at weaning. Piglets were weaned at an average age of 19.6 d (range 11 to 28 d).

### Experiment 3

This trial took place at the same commercial sow farm as described for Exp. 2, in May–July, 2016. The protocol used was the same as that described for Exp. 2, except that the dose of glucose and glucosamine was increased to 20 g/d, to account for the larger sows compared to the gilts in Exp. 1. In addition, only parity 3 to 7 sows were treated, no parity 2 sows were available within the management system at the time of the trial. In this experiment, 89 sows received glucosamine and 87 sows received glucose. As in Exp. 2, piglets were ear tagged and weighed and then crossfostered after birth but it was not possible to record crossfostering until piglets were weighed at weaning.

### Statistical analysis

Fetal number (uterine capacity) data from Exp. 1 was analyzed using PROC MIXED (SAS Inst. Inc., Cary, NC) with a model that included treatment. Fetal blood glucose and fructose were analyzed with a model that included treatment, fetal weight, placental weight, and treatment by fetal weight and treatment by placental weight interactions. Gilt within treatment was included as a random effect. Interaction terms that were not significant were sequentially dropped from the model to arrive at a final model. Placental morphometry and hyaluronan data were analyzed using a model that included effects of treatment, fetal size and the treatment by size interaction. Gilt within treatment was included as a random effect. Relationships between placental hyaluronan and fetal serum glucose and fructose for the corresponding largest and smallest fetuses were analyzed using a model that included treatment, fetal size, the treatment by fetal size interaction, fetal weight, fetal weight by treatment, fetal weight by fetal size, fetal weight by treatment by fetal size, placental weight, placental weight by treatment, placental weight by fetal size, and placental weight by treatment by fetal size. Nonsignificant effects were sequentially removed from the model, starting with complex interactions, until only significant effects remained.

Litter size data from Exp. 2 and 3 were analyzed using PROC MIXED with a model that included the effects of farrowing week, treatment, parity and the treatment by parity interaction. Orthogonal contrasts were used when necessary to further evaluate differences among treatment means. Birth and weaning weights were considered repeated measures of the birth dam and were therefore analyzed with a similar model to litter size data, including effects of farrowing week, treatment, parity and the treatment by parity interaction. Sow within week by treatment by parity interaction was included as a random effect. Finally, stillbirth rate and preweaning mortality were also considered a trait of the birth dam and analyzed using PROC GLIMMIX, treating alive or dead at birth and weaning as binary variables. Because it was not possible to record crossfostering, crossfostering was considered to be random and was not considered in the analysis of preweaning mortality. Thus, this analysis treats weaning weights and preweaning mortality as traits of the birth dam, not traits of the lactating dam. The model included effects of farrowing week, treatment, parity and treatment by parity, and the effect of sow within week by treatment by parity was included as a random effect.

## Results

### Experiment 1

Number of CL for glucosamine- and glucose-treated UHO gilts did not differ (16.2 ± 0.9 and 15.1 ± 0.9, respectively). There was a trend (*P* = 0.098) toward greater number of live fetuses in UHO gilts treated with glucosamine compared to glucose (8.4 ± 0.6 and 6.9 ± 0.6, respectively).

**Fig. 1:**
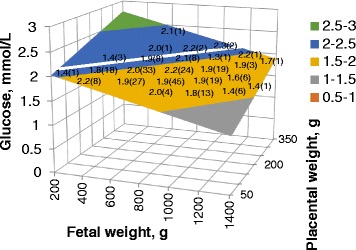
Effect of fetal and placental weight on fetal serum glucose. Both fetal weight and placental weight affected fetal serum glucose concentrations, with no effect of glucosamine supplementation (glucose = 2.06–0.00072(fetal weight) + 0.002401(pwt); *P* < 0.05 for both effects). The white line indicates where the effects of fetal and placenta weights on glucose balance, yielding no change in glucose concentrations. This occurs at a ratio of placental weight to fetal weight of 0.30. Means with number of observations are included at positions within the surface plot to reflect how well the surface plot corresponds with the actual observations

There were no treatment interaction effects on the relationships between fetal weight and fetal serum glucose and placental weight and fetal serum glucose, nor was there an overall effect of treatment on fetal serum glucose when interaction effects were removed from the model. However, both fetal weight and placental weight were associated (*P* < 0.05) with fetal serum glucose, and these relationships are illustrated in Fig. 1. The surface plot in Fig. 1 indicates that fetal serum glucose decreased with increasing fetal weight and increased with increasing placental weight (fetal glucose = − 0.0072 (fetal weight) + 0.002401 (placental weight)). The two effects balanced (no net effect on glucose concentrations) when placental weights were 30% of fetal weights (white line indicated in Fig. 1).

**Fig. 2:**
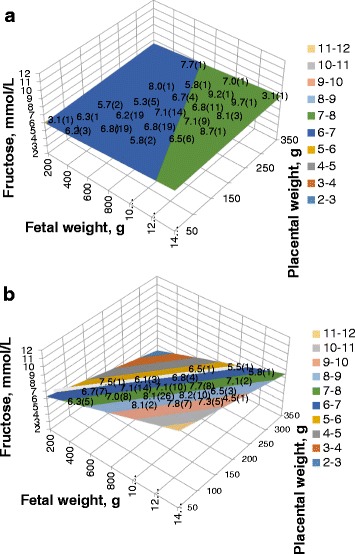
Effect of fetal and placental weight on fetal serum fructose-glucosamine treated (**a**), glucose treated (**b**). Response surfaces differed between treatments (fructose = 5.6916 + 0.00098(fetal weight) + 0.00185(placental weight) for glucosamine supplemented gilts; fructose = 6.1504 + 0.00438(fetal weight) – 0.01363(placental weight); slope for fetal weight *P* = 0.09; slope for placental weight *P* < 0.05). Within glucose-treated gilts, the white line indicates the fructose concentration where the effects of placental and fetal weight balance, which is at a ratio of 0.32. Means with number of observations are included at positions within the surface plots to reflect how well the surface plots corresponds with the actual observations

Fetal serum fructose relationships with fetal and placental weights were affected by treatment, and the effects are illustrated in Fig. 2. The final model included significant treatment by fetal weight (*P* < 0.01) and treatment by placental weight (*P* < 0.05) interaction terms. In glucosamine-treated gilts, fetal serum fructose was positively related to both fetal weight and placental weight (fetal serum fructose = 5.6916 + 0.00098 (fetal weight) + 0.00185 (placental weight)). In glucose-treated gilts, fetal serum fructose was positively related to fetal weight and negatively related to placental weight (fetal serum fructose = 6.1504 + 0.00438 (fetal weight) – 0.01363 (placental weight)). Similar to relationships between fetal weight, placental weight and fetal serum glucose, the effects of fetal weight and placental weight on fetal serum fructose in glucose treated gilts balanced when placental weight was 32% of fetal weight (white line in Fig. 2a). Few live fetuses were present in the dataset where the placenta was greater than 30% of fetal weight at d 105 of gestation.

**Fig. 3:**
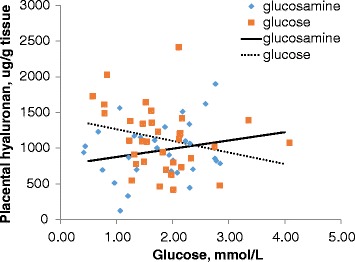
Effect of glucose on hyaluronan by treatment. Heterogeneity of regression indicated that the two lines differed (glucosamine supplemented: hyaluronan = 760.44 + 115.77(glucose); glucose supplemented: hyaluronan = 1427–162.64; *P* < 0.05 for intercept; *P* = 0.06 for slope)

Fetal and placental weight, fetal blood glucose and fructose, and placental hyaluronan for the largest and smallest fetuses in each litter are summarized in Table 1. Hyaluronan data from the largest and smallest placentas from one gilt were unusually high (> 3 standard deviations above the mean), and were therefore deleted as outliers. There was no effect of treatment or treatment by fetal size interaction on these traits. Fetal and placental weights were significantly reduced (*P* < 0.01) in the smallest compared to the largest fetuses in the litter. Despite results from all fetuses within the litter indicating that glucose decreased with fetal weight, there was no significant effect of fetal size on fetal serum glucose in this reduced dataset. Similar to results from all fetuses in the litter, there was a significant decrease (*P* < 0.05) in fructose in the smallest fetus compared to the largest fetus in the litter. Placental hyaluronan was unaffected by fetal size as a category, or by fetal weight or placental weight as continuous variables. Further exploration of relationships between hyaluronan and fetal serum glucose and fructose using regression analysis indicated that the relationship between hyaluronan and serum fructose concentrations was unaffected by treatment or fetal size and that there was no overall relationship. The relationship between hyaluronan and fetal serum glucose did not differ between large and small fetuses, but did differ between treatments (treatment intercepts, *P* < 0.05; treatment slopes, *P* = 0.06; Fig. 3). Placental hyaluronan was positively related to glucose in glucosamine-treated gilts, and negatively related to glucose in glucose-treated gilts.

**Table 1:** Treatment effects on fetuses from Experiment 1

	Glucosamine^a^		Glucose	
Variable	Large	Small	Large	Small
Fetal weight, g^b^	1017 ± 52	529 ± 52	1076 ± 49	639 ± 49
Placental weight, g	231 ± 13	108 ± 13	219 ± 13	129 ± 13
Serum glucose, mmol/L	1.8 ± 0.2	1.7 ± 0.2	1.9 ± 0.2	1.7 ± 0.2
Serum fructose, mmol/L^c^	6.7 ± 0.6	5.6 ± 0.6	7.7 ± 0.5	6.7 ± 0.5
Placental hyaluronan, mg/g tissue^d^	1.06 ± 0.11	0.87 ± 0.11	1.08 ± 0.10	1.18 ± 0.10

^a^Number of observations is 16 for glucosamine and 17 for glucose. Least squares means for fetal weight, placental weight, serum glucose and fructose and placental hyaluronan for the smallest and largest fetus in glucosamine and glucose-treated gilts from Exp. 1 are presented

^b^Effect of fetal size (*P* < 0.01)

^c^Effect of fetal size (*P* < 0.05)

^d^Placental hyaluronan results from one sow with very high hyaluronan (> 3 standard deviations above mean) were deleted from the analysis

The results of placental morphometry are summarized in Table 2. Placental fold width was significantly greater (*P* = 0.05) in glucosamine-treated gilts compared to glucose-treated gilts. There was no effect of size of the fetus on placental fold width, and there was no treatment by size of fetus interaction. Stromal depth above the folded bilayer did not differ with treatment or size of fetus, but there tended to be a treatment by fetal size interaction (*P* = 0.07). This appeared to be due to greater stromal depth in large fetuses from glucosamine-treated UHO gilts compared to the other three treatment by fetal size combinations (*P* < 0.01). The total width of the placenta was greater (*P* < 0.05) in glucosamine-treated gilts compared to glucose-treated gilts, and there was no effect of fetal size on total placental width nor was there a treatment by fetal size interaction. Finally, the length of the folded bilayer adjusted to a constant length of placenta was unaffected by treatment, fetal size or the treatment by fetal size interaction. Nevertheless, the adjusted length of the folded bilayer was significantly correlated (*r* = 0.75; *P* < 0.01) with the width of the folded bilayer.

**Table 2:** Treatment effects on placental morphometry from Experiment 1

	Glucosamine^a^		Glucose	
Variable	Large	Small	Large	Small
Fold Width, μm^b^	778 ± 37	818 ± 37	716 ± 36	731 ± 36
Stromal Width, μm^c^	236 ± 28	142 ± 28	140 ± 27	146 ± 27
Total Width, μm^b^	1014 ± 47	961 ± 47	856 ± 46	876 ± 46
Interface length/unit placental length^c^	7470 ± 403	7546 ± 403	6969 ± 389	6727 ± 389

^a^Number of observations is 16 for glucosamine and 17 for glucose. Least squares means for bilayer fold width, stromal width above the folded bilayer, total placental width and placental bilayer interface length per unit placental length from Exp. 1 are presented

^b^Effect of treatment (*P* ≤ 0.05)

^c^Effect of treatment by fetal size (*P* = 0.07)

### Experiment 2

There was a treatment by parity interaction for number of stillborn piglets and stillbirth rate. No treatment by parity interaction was observed for any of the other traits measured in this experiment. The treatment by parity least squares means for number of stillborn piglets and stillbirth rate are presented in Table 3. For both number of stillborns and stillbirth rate, the interaction appeared to be due to greater stillbirth in glucosamine supplemented sows in later parities (parities 7 and 8).

**Table 3:** Treatment by parity effect on number stillborn and stillbirth rate from Experiment 2

	Treatment			
Parity^a^	Glucosamine		Glucose	
	Number stillborn	Stillbirth rate	Number stillborn	Stillbirth rate
2	1.0 ± 0.3 (20)^b^	0.06 ± 0.02	0.8 ± 0.3 (20)	0.06 ± 0.02
3	1.4 ± 0.3 (30)	0.08 ± 0.02	1.0 ± 0.3 (30)	0.07 ± 0.01
4	0.9 ± 0.4 (15)	0.06 ± 0.02	1.3 ± 0.4 (16)	0.07 ± 0.02
5	1.8 ± 0.4 (14)	0.09 ± 0.02	1.8 ± 0.5 (11)	0.11 ± 0.03
6	1.8 ± 0.4 (14)	0.10 ± 0.03	2.2 ± 0.4 (15)	0.11 ± 0.03
7	2.1 ± 0.3 (23)	0.14 ± 0.03	1.5 ± 0.3 (23)	0.10 ± 0.02
8	2.6 ± 0.4 (12)	0.22 ± 0.04	1.2 ± 0.4 (12)	0.09 ± 0.03

^a^For both traits, the interaction contrast comparing the interaction between treatments and parities 2 through 6 combined versus parities 7 and 8 combined was statistically significant (*P* < 0.05), indicating that more stillborn piglets occurred in the glucosamine-treated parity 7 and 8 sows compared to earlier parity sows. Least squares means for the treatment by parity interaction for number of stillborn piglets and stillbirth rate from Exp. 2 are presented

^b^Numbers of observations are in parentheses

Treatment least squares means for the other litter size and weight traits are presented in Table 4. There were no statistically significant effects of treatment on the number of total born, born alive, or mummies, or on birth weights, weaning weights, or preweaning mortality.

**Table 4:** Treatment effects on litter traits from Experiment 2

	Treatment^a^	
Variable	Glucosamine	Glucose
Total born	15.6 ± 0.4 (128)	15.2 ± 0.4 (127)
Born alive	14.0 ± 0.3	13.8 ± 0.3
Mummies	0.56 ± 0.08	0.57 ± 0.08
Birth weights	1.37 ± 0.02 (1970)	1.35 ± 0.02 (1915)
Weaning weights	5.48 ± 0.06	5.43 ± 0.06
Preweaning mortality	0.17 ± 0.01	0.16 ± 0.01

^a^Differences between glucosamine and glucose supplementation were not statistically significant. Number of observations are in parentheses. Litter size trait, birth and weaning weight least squares means for glucosamine and glucose supplemented sows from Exp. 2 are presented

Significant parity effects (Table 5) were observed for the number of total born (*P* < 0.05) and live born piglets (*P* < 0.05), and for birth (*P* < 0.01) and weaning weights (*P* < 0.05). Number of mummies and preweaning mortality were not affected by parity. Total born and born alive increased gradually with increasing parity until parity 6, after which both litter size measures decreased. Birth weights were similar among parities until parity 6, after which they also decreased (*P* < 0.05). In contrast, average weaning weights increased progressively with increasing parity.

**Table 5:** Parity effects on litter traits from Experiment 2

	Parity						
Variable	2	3	4	5	6	7	8
Total born^a^	14.7 ± 0.6(40)^b^	15.3 ± 0.5(60)	15.5 ± 0.7(31)	16.4 ± 0.8(25)	17.3 ± 0.7(29)	14.4 ± 0.6(46)	14.5 ± 0.8(24)
Born alive^a^	13.8 ± 0.6	14.1 ± 0.5	14.4 ± 0.6	14.6 ± 0.7	15.3 ± 0.7	12.6 ± 0.5	12.6 ± 0.7
Mummies	0.29 ± 0.14	0.59 ± 0.12	0.64 ± 0.16	0.53 ± 0.18	0.72 ± 0.17	0.69 ± 0.13	0.48 ± 0.18
Birth weights^c^	1.45 ± 0.04	1.45 ± 0.03	1.40 ± 0.04	1.39 ± 0.04	1.33 ± 0.04	1.34 ± 0.03	1.33 ± 0.05
Weaning weights^d^	5.29 ± 0.09	5.30 ± 0.08	5.34 ± 0.12	5.38 ± 0.12	5.57 ± 0.11	5.67 ± 0.09	5.64 ± 0.13
Preweaning mortality	0.14 ± 0.02	0.15 ± 0.02	0.17 ± 0.02	0.18 ± 0.02	0.16 ± 0.02	0.22 ± 0.02	0.19 ± 0.03

^a^Contrasts indicated a progressive increase to parity 6, followed by a precipitous decrease (*P* < 0.05). Litter size trait, birth and weaning weight least squares means for second through eighth parity sows from Exp. 2 are presented

^b^Number of observations are in parentheses

^c^Contrasts indicated no differences among parities 2 through 6, and a decrease in parities 7 and 8 (*P* < 0.01)

^d^ Contrasts indicated a progressive increase from parity 2 to 8 (*P* < 0.05)

### Experiment 3

There was an overall effect of treatment (*P* < 0.05; Total piglets born 17.9 ± 0.5 for glucosamine, 16.5 ± 0.5 for glucose; Number born alive 15.9 ± 0.4 for glucosamine, 14.6 ± 0.4 for glucose) and a treatment by parity interaction for total piglets born and piglets born alive (*P* < 0.01; Table 6). Orthogonal contrasts indicated that glucosamine treatment resulted in greater total piglets born and born alive in later parities (5 and 6) compared to early parities and parity 7. There were no effects of treatment on the number of stillbirths or mummies. Analysis of birth and weaning weights indicated that glucosamine had no effect on weights compared to glucose treatment. Finally, there was no difference in preweaning mortality between the two treatment groups.

**Table 6:** Treatment by parity effects on litter traits from experiment 3

Treatment	Total born^a^	Born alive^a^	Stillborns	Mummies	Birth weight	Weaning weight	Preweaning mortality
Glucosamine							
Parity 3	17.9 ± 0.7	15.9 ± 0.7	1.3 ± 0.3	0.8 ± 0.2	1.40 ± 0.04	5.0 ± 0.1	0.11 ± 0.02
Parity 4	16.8 ± 0.9	14.9 ± 0.8	1.3 ± 0.3	0.5 ± 0.2	1.41 ± 0.05	4.9 ± 0.1	0.15 ± 0.02
Parity 5	20.0 ± 1.2	17.2 ± 1.0	1.7 ± 0.5	1.1 ± 0.3	1.26 ± 0.06	5.1 ± 0.2	0.16 ± 0.03
Parity 6	18.7 ± 1.2	17.2 ± 1.1	0.5 ± 0.5	1.0 ± 0.3	1.32 ± 0.07	5.1 ± 0.2	0.17 ± 0.03
Parity 7	16.2 ± 1.0	14.3 ± 0.9	1.5 ± 0.4	0.3 ± 0.2	1.37 ± 0.06	5.2 ± 0.2	0.11 ± 0.02
Glucose							
Parity 3	17.6 ± 0.8	16.0 ± 0.7	1.0 ± 0.3	0.6 ± 0.2	1.36 ± 0.04	5.1 ± 0.1	0.11 ± 0.03
Parity 4	17.9 ± 0.9	15.8 ± 0.8	1.5 ± 0.4	0.6 ± 0.2	1.41 ± 0.05	4.8 ± 0.2	0.18 ± 0.03
Parity 5	15.8 ± 1.1	14.7 ± 1.0	0.9 ± 0.4	0.2 ± 0.3	1.42 ± 0.06	4.8 ± 0.2	0.12 ± 0.03
Parity 6	15.1 ± 1.1	12.1 ± 1.0	2.4 ± 0.4	0.6 ± 0.3	1.30 ± 0.06	5.0 ± 0.2	0.24 ± 0.04
Parity 7	15.8 ± 1.1	14.3 ± 0.9	1.1 ± 0.4	0.5 ± 0.3	1.41 ± 0.06	4.8 ± 0.2	0.08 ± 0.02

^a^A treatment by parity interaction was present. Orthogonal contrasts indicated that total born and born alive were greater in later parities (5 and 6) in glucosamine treated sows compared to glucose treated sows, but were not different in early parities or in parity 7. Litter size trait, birth and weaning weight least squares means for glucosamine- and glucose-supplemented sows for each parity from Exp. 3 are presented

## Discussion

Results indicated that glucosamine supplementation tended to improve the number of live fetuses in UHO gilts (a measure of uterine capacity), altered the microscopic architecture of the developing pig placenta, and changed relationships between fetal size, fetal serum glucose and fructose, and placental hyaluronan. In Exp. 2, 10 g/d glucosamine supplementation of intact sows in a commercial herd did not result in a beneficial effect at the dose used in gilts. One possible contributing factor to the lack of treatment effect on the number of piglets born alive in Exp. 2 was an increased number of stillborn piglets in late parity sows treated with glucosamine. Comparisons of total born, born alive and birth weights among parities confirmed that reductions in each occurred in parity 7 and 8 sows, so it is possible that some interaction between the reproductive competence of late parity sows and glucosamine supplementation may explain the increase in stillbirth incidence. Because the dose used in Exp. 2 may not have been sufficient in the larger sows to observe an effect on litter size, the trial was repeated in Exp. 3 using 20 g/d. Results of Exp. 3 indicate a substantial (+1.3 piglets born alive overall) gain in litter size compared to glucose treatment and the effect was greater in later parity sows.

Glucose and fructose are part of the pathway that results in glucosamine synthesis [15]. In Exp. 1, serum glucose and fructose concentrations were measured to determine whether they were altered by glucosamine supplementation. Results indicated significant relationships for both glucose and fructose with fetal and placental weights. Glucose concentrations were negatively related to fetal weight and positively related to placental weight. These relationships are consistent with the concept that within the conceptus, glucose originates from the placenta and is used by the fetus [16, 17]. These relationships were unaffected by treatment. In contrast, fetal and placental weight relationships with fructose concentrations were affected by treatment. Glucose treated gilts may be considered the “normal” fetal and placental weight relationships with fructose because a daily dose of 10 or 20 g glucose added to a diet of 2 kg/d of a corn-soy diet would not be expected to add much available glucose to the gestating gilt (the corn-soybean based daily diet contains at least 1 kg starch, which would be converted to glucose during digestion). Curiously, in glucose-treated gilts at d 105 of gestation, fetal plasma fructose was positively related to fetal weights and negatively related to placental weights. Previous reports indicate that both the placenta and the fetus are sources of fructose during gestation [18, 19]. Results from glucose supplemented gilts suggest that at d 105 of gestation, the placenta may be a net consumer of fructose and the fetus may be a net producer of fructose. Glucosamine supplementation resulted in reducing the positive relationship with fetal weight, and converting the negative relationship with placental weight to a positive one, essentially stabilizing fructose concentrations as fetal and placental weights vary (Fig. 2). These changes could be consistent with the role of fructose in the synthesis of glucosamine [15]. Thus, Fig. 2 is consistent with the concept that providing exogenous glucosamine might reduce fructose use by the placenta and stabilize fructose concentrations.

Hyaluronan is a major component of the placental stroma [7, 8]. Glucosamine is a major component of hyaluronan, the other component being glucuronic acid [9]. The folded bilayer interface undergoes extensive remodeling during late gestation (d 85 onward), characterized by increased stromal development between individual folds and development of secondary folds [7, 13]. These processes likely require both synthesis of new hyaluronan and turnover of existing hyaluronan. This is likely to require the synthesis of both glucuronic acid and glucosamine, which both originate from glucose [20], although fructose is an intermediate in glucosamine synthesis [15]. Paradoxically, glucosamine supplementation altered the relationship between serum glucose and placental hyaluronan, and no relationship between fructose and hyaluronan was demonstrated, despite fructose being an intermediate in glucosamine synthesis. However, no difference in placental hyaluronan occurred between treatments. It is possible that glucosamine altered the relationship between hyaluronan and glucose by improving turnover without affecting fructose, which may have made the placenta more efficient at glucose transport, resulting in a positive relationship. This possible mechanism would be supported by changes in the microscopic architecture of the placental folds.

Results indicated increased total thickness of the placenta, increased width of the folded bilayer, and increased stromal depth above the folded bilayer in placenta of large fetuses. These changes are all consistent with the concept that glucosamine encourages stromal development, and that this in turn provides the substrate needed to encourage folded bilayer development. Given our previous hypothesis [7], one would expect improvement in placental transport of nutrients. However, if nutrient transport was improved, one might expect an increase in fetal weight, especially for small fetuses. No change in the weight of the smallest fetus was observed. On the other hand, it is possible that there is a threshold weight of the smallest living fetus for fetal survival. If this is true, the weight of the smallest living fetus might be unchanged, but more fetuses would survive because more fetuses would be above the survival threshold as a result of improved transport. Thus, the trend in greater litter size in glucosamine-treated gilts observed in this experiment supports the hypothesis that the improvements in fold development resulted in improved nutrient transport that then increased litter size.

The UHO surgical procedure removes one ovary and one uterine horn, and the remaining ovary undergoes compensatory hypertrophy such that ovulation rate is unaffected. This results in the same number of available embryos in half the uterine space, and litter size in UHO gilts is considered to be a direct measure of one-half uterine capacity [5]. Results of experiment 1 indicated a numerical increase in litter size of 1.4 live fetuses on 105 d of gestation, so it was conceivable that uterine capacity was increased by nearly 3 live fetuses. The UHO measure was uncomplicated by the incidence of stillbirth, because the measure was made at slaughter on d 105 of gestation. Previous selection for uterine capacity using the UHO model resulted in an increase in uterine capacity of approximately one fetus per uterine horn, and a significant increase in litter size in intact selected gilts, although the effect on litter size was less due to the influence of ovulation rate on litter size in intact gilts [21]. Nevertheless, given the results of Exp. 1, it seemed likely that glucosamine would increase litter size in intact pigs, thus warranting a larger test in intact sows.

Results of Exp. 2 indicated no significant effect of 10 g/d of glucosamine supplementation on litter size in sows ranging in parity from 2 to 8 in contrast to the results of Exp. 1 in which a trend toward improved litter size was obtained. In Exp. 2, we used the same dose of glucosamine for sows as for the gilts in Exp. 1. However, because they were not as old, the gilts in Exp. 1 weighed less than the sows used in Exp. 2. To explore whether a greater dose would be effective, we performed a second commercial trial using 20 g/d glucosamine. Results of Exp. 3 indicated that a dose of 20 g/d in sows was effective in increasing both the total number of piglets born and the number born alive with greater increases occurring in later parity (5 and 6) sows. Analysis of birth and weaning weights indicated no change in piglet weights despite the increase in litter size of greater than 1 piglet per litter; therefore, the increase in litter size occurred without a depression in birth weights. Finally, there was no difference in preweaning mortality, indicating that the increase in litter size should have resulted in an increase in the number of weaned piglets.

An obvious complicating factor in Exp. 2 and 3 is the ovulation rate of the sows used, which we were not able to measure because of the commercial setting. Previous observations of ovulation rates in commercial sows have indicated very high ovulation rates, particularly in later parity sows [22]. High ovulation rates in parity 5 and 6 sows may explain the interaction effect observed. If similar ovulation rates to those previously reported occurred in the parity 5 and 6 sows used in our commercial trials, they are likely to have been sufficient to provide a good test of uterine capacity in intact females. Ovulation rates in earlier parity animals may not have been sufficient, resulting in the interaction observed. The commercial farm in this experiment obtains maternal line gilts from DNA genetics (Columbus, NE; formerly Danbred USA), but we could find no published estimates of ovulation rates in these maternal line gilts. However, there are published litter size estimates for Danish maternal line gilts, demonstrating excellent genetic progress in litter size selection [23], and it is likely that high ovulation rates contributed to the reported increase in litter size due to selection. Thus, ovulation rates were likely to be high enough in the parity 5 and 6 sows to put greater emphasis on uterine capacity as a determining factor for litter size. Exception to this may have been the late parity sows in Exp. 2 and 3 (parity 7 and 8). In Exp. 2 these sows clearly had reduced numbers of total born and born alive piglets, and one contributing factor to this decrease could have been reduced ovulation rates. We could not find any published reports of ovulation rates specifically in sows in later parities for commercial herds, primarily because previous reports combined ovulation rate estimates of parities 4 or greater [22]. Thus, whether ovulation rate decreased in parity 7 and 8 sows under some conditions, resulting in lower litter size, remains unknown.

The increase in the number of stillborn piglets and stillbirth rates in parity 7 and 8 sows supplemented with glucosamine in Exp. 2 was an unexpected result. Results of Exp. 1 indicated that glucosamine supplementation increased the depth of the folded bilayer in the pig placenta regardless of the size of the fetus, and because of this the total width of the placenta was increased. Van Rens and Van der Lende [24] implicated a thicker placenta in prolongation of individual piglet birth intervals, and suggested that placental thickness may contribute to stillbirth rate due to the well-known relationship between piglet birth intervals and stillbirth [25]. Van Rens and Van der Lende [24] suggested that thicker placentas may present a greater barrier to delivery of the piglet during farrowing, increasing birth intervals. However, the effective width of the placenta is likely to be the width of the stroma above the folded bilayer, which we have reported to be reduced in small fetuses compared to large fetuses [7]. Results of Exp. 1 confirm that the stroma above the folds is greater in placenta of large fetuses in gilts supplemented with glucosamine. The incidence of larger fetuses, and therefore thicker placenta, might be expected to increase with decreasing litter size. It is possible that the reduced litter size in parity 7 and 8 sows observed in Exp. 2 resulted in thicker placenta in these sows upon glucosamine supplementation, which could have prolonged birth intervals (which we did not measure), and increased stillbirth. Whatever the mechanism for the increase in stillbirth rate, results from Exp. 2 suggest that glucosamine supplementation may be detrimental in parity 7 and 8 sows due to increased stillbirth rate.

## Conclusions

Maternal supplementation with glucosamine altered fetal serum glucose and fructose dynamics and increased the depth of the placental folded bilayer in placenta of UHO gilts. Results are consistent with the concept that glucosamine supplementation may have stabilized fetal fructose concentrations as fetal and placental weights vary among conceptuses. The placental epithelial bilayer fold-depth changes occurred with a trend toward increased uterine capacity of about 1.4 fetuses per uterine horn measured in UHO gilts. In commercial trials, glucosamine supplementation in intact sows resulted in a slight numerical but nonsignificant increase in the number of piglets born and born alive at a dose of 10 g/d glucosamine and a significant increase in litter size of greater than 1 piglet per litter at a dose of 20 g/d. The increased litter size occurred in later parity 5 and 6 sows. Thus, it is likely that 10 g/d glucosamine was not sufficient in commercial sows due to their larger size compared to the gilts in Exp. 1. The increase in litter size from a dose of 20 g/d occurred without reductions in birth or weaning weights and with no reduction in preweaning mortality. Our results further indicated an increase in the number of stillborn piglets and stillbirth rate in glucosamine-treated late parity sows (parity 7 and 8) in Exp. 2, which was unexpected. This may have been due to the effect of glucosamine supplementation on placental thickness, which could have occurred due to the reduced litter sizes in these late parity sows. These results suggest that glucosamine supplementation at a dose of 20 g/d could be useful in increasing litter size in commercial parity 5 and 6 sows, resulting in increased number of piglets weaned. Because the mechanism of the increase is likely to be based on improved placental function and uterine capacity, an increase would only be expected if ovulation rates are sufficient.

## Abbreviations

GLUT: Glucose transporter
UHO: Unilaterally hysterectomized ovariectomized
USMARC: US Meat Animal Research Center
